# Prevalence, Patterns, and Factors Associated with Peripheral Neuropathies among Diabetic Patients at Tertiary Hospital in the Kilimanjaro Region: Descriptive Cross-Sectional Study from North-Eastern Tanzania

**DOI:** 10.1155/2019/5404781

**Published:** 2019-06-04

**Authors:** Ahlam A. Amour, Nyasatu Chamba, Johnstone Kayandabila, Isaack A. Lyaruu, Dekker Marieke, Elichilia R. Shao, William Howlett

**Affiliations:** ^1^Department of Internal Medicine, KCMUCo, Moshi, Tanzania; ^2^Department of Internal Medicine, KCMC Hospital, Moshi, Tanzania; ^3^Mnazi Mmoja Hospital, Zanzibar, Tanzania; ^4^Image Doctors Organization, Arusha, Tanzania; ^5^Better Human Health Foundation, Moshi, Tanzania

## Abstract

**Objective:**

Diabetic peripheral neuropathy (DPN) is a common microvascular complication of diabetes mellitus (DM) and may progress to diabetic foot, which frequently leads to amputation and/or disability and death. Data is scanty on the burden of diabetic peripheral neuropathy in Tanzania. The aim of this study was to assess the burden of peripheral neuropathy, its severity, and the associated factors.

**Methods:**

The study was a cross-sectional hospital-based study and was carried out from October 2017 to March 2018 among adolescent and adult patients attending Kilimanjaro Christian Medical Center (KCMC) diabetes clinic.

**Results:**

A total of 327 diabetic patients, females n=215 (65.7%) and males n=121 (34.3%), were included in the study. The mean age was 57.2 yrs. A total of 238 (72%) had type 2 and 89 (27.2%) had type1 DM. The prevalence of peripheral neuropathy was 72.2% of whom 55% were severe, 19% were moderate, and 26% were mild. The severity of neuropathy increased with the increase in age >40 years (p < 0.001) and increase in body mass index (p<0.001) and duration of diabetes; duration >7 years (p <0.006). The main associated factors were age >40 years, OR 2.8 (1.0-7.7), >60 years, OR 6.4 (2.3-18.2), obesity, OR 6.7 (0.9-27.7), and hypertension, OR 4.3 (2.2-8.2).

**Conclusion:**

More than half of the patients included in this study were found to have neuropathy, nearly half of whom presented with the severe form. The main risk factors were increasing age, increasing duration of diabetes, obesity, and hypertension. Diabetic peripheral neuropathy is underdiagnosed in northern Tanzania where screening for neuropathy is not routinely done.

## 1. Introduction

Diabetes mellitus (DM) is a major health problem globally [1]. The global prevalence is 8.8% among adults with the number expected to rise to 10.4% by 2040 [2]. Type 2 DM accounts for 90–95% of all diagnosed cases of diabetes with higher prevalence among older adults [3, 4]. Diabetes and its complications are rapidly becoming the world's most significant cause of morbidity and mortality. It is estimated that around 360 million patients globally will have DM by 2030. The two main complications affecting limbs, mainly feet and legs, are diabetic polyneuropathy (DPN) which affects between 30 and 50% of diabetics and diabetic leg and foot ulcers. The lifetime incidence of foot ulcers occurring in DM patients is up to 25% [4, 5]. Diabetic neuropathy is the primary risk factor for the development of diabetic foot ulcers [6] and is implicated in 50–75% of nontraumatic amputations. 

It is estimated that approximately 50% of diabetics suffer from DPN, [7] and in 50% of these it is at least of moderate severity. [8–10]. The frequency of DPN in DM varies widely from 9.6 to 88.7% globally. This might be due to different types of diabetes, disease duration, existing healthcare facilities, sample selection, different diagnostic criteria used, and variable methods used in physical examination [1, 11–16]. DPN when present is mainly irreversible; hence screening and identifying associated potentially modifiable risk factors is very crucial especially for the low-income countries. The main risk factors that are known to be associated with DPN are increasing age, longer duration of diabetes since diagnosis, poor glycemic control, and increased body mass index. However, data in Tanzania on the frequency and the associated risk factors for DPN is scanty, hence difficulties in implementing prevention, modification, and treatment plans. This study will evaluate the prevalence of DPN and its associated risk factors with the aim to reduce the enormous medical and socioeconomic burden.

## 2. Methods

A prospective hospital based cross-section study was conducted from October 2017 to March 2018 at Kilimanjaro Christian Medical Center (KCMC) referral and teaching hospital in Northern Tanzania. The hospital has 640 beds with outpatient clinics and special clinics for diabetes and endocrine disorders. It has a catchment area with a population of >15million and nearby population from our neighboring country Kenya. Patient recruitment was done in two different weekly diabetic clinics, adolescents, 14–22 years, and adults >22 years.

### 2.1. Participants

The study population included patients with type one or two DM aged 14 years and above. Diabetic patients presenting with HIV or tuberculosis on treatment or chemotherapy were excluded based on medical history hospital notes and baseline screening tests. A minimum sample size was calculated (n=315) based on estimated frequency of DSN. Using systemic random sampling technique to recruit participants who met the inclusion criteria every 8th patient file was taken, and patient was interviewed and examined until the minimal sample size was reached.

### 2.2. Study Procedures

A standardized questionnaire was used to collect the social-demographic data, disease associated information, and clinical characteristics. Patients were diagnosed for neuropathy by using the Toronto Clinical Scoring System (TCSS) tool, which consists of three parts. The first part is history version that is included in the questionnaire and the last two parts (second and third) contain physical assessment examination performed by the principal investigator.

The clinical tests from TCSS score carries were as follows: Pressure sensation was assessed using 10gm monofilament at 10 standard sites of the sole of the feet, pain sensation was done using a pin-prick, vibration sense was tested by using a 128-Hz tuning fork which was put on the first toe at bony prominent area, and temperature was tested using cylinders with different temperatures (cold and warm) placed on the dorsum of the foot. Tendon reflex was tested by striking the Achilles and quadriceps tendons with a reflex hammer. The anthropometric measurements included were height (meters) and weight (kg) and these were measured to calculate body mass index (BMI) in kg/m2. Normal weight was defined as BMI of 18.5 to 24.9 kg/m^2^, while overweight and obesity were defined as BMI of 25 to 29.9 kg/m^2^ and ≥30 kg/m^2^, respectively. A standard procedure was used to measure the blood pressure (BP) of participant using the right arm with a manual sphygmomanometer. Hypertension was defined based on Joint National Committee 7 (JNC 7) as systolic blood pressure ≥ 140 mmHg, and diastolic blood pressure ≥ 90 mmHg, or with antihypertensive treatment [17]. Blood samples for hemoglobin (Hb) A1C and creatinine were analyzed using COBAS INTEGRA 400® PLUS (Roche Diagnostics Ltd, CH-6343 Rotkreuz, Switzerland) Analyzer, and this was done by the laboratory technician. The normal HbA1C was ≤ 6.8%. Glucoplus™ (Glucoplus Inc, Saint Laurent, QC H4S 1S3 Canada) meter was used for assessment of patient's fasting blood glucose/random blood glucose. This was done by a nurse. Lastly the principal investigator assessed for lower-extremity peripheral neuropathy using a TCSS score (see Appendix). The TCSS consists of 6 clinical symptoms, 5 sensory tests and lower limbs reflexes, which give a maximal score of 19. Severity of neuropathy was classified based on the score as no neuropathy (0–5), mild neuropathy (6–8), moderate neuropathy (9–11), and severe neuropathy ≥ 12. The tests were applied on the patient's hand prior to the examination of the foot and the patient was asked to close the eyes during examination.

### 2.3. Statistical Analysis

Data were coded and entered using Excel and explored to SPSS version 22. Missing values and data cleaning were clearly checked. Data were examined for distribution and outliers, through univariable analysis. Descriptive analysis was completed generating means, medians, standard deviations, and interquartile ranges for quantitative data and frequency distributions for categorical data. Student's t-test was used to compare the difference in means for continuous variables, while Chi-Square test was used to compare proportions of categorical variables. The Odds ratios (ORs) with 95% confidence intervals (CIs) for prevalence of peripheral neuropathy and associated factors among diabetic patients were estimated using multivariable logistic regression model while controlling for potential confounders. A variable was considered to be a confounder if its inclusion in the model changed the crude odd ratio by 10% or more.* P* values less than or equal to 0.05 were considered statistically significant, using a two-sided test of hypothesis.

Informed written consent (by signature or thumbprint) was obtained from all participants. Ethical clearance (No 2087) was sought and granted by the Institution Review Board at KCMU-College and Ethical Committee, and permission was obtained from Internal Medicine Head of Department and Adolescent Diabetic Clinic in charge before commencing the study.

## 3. Results

A total of 338 patients were enrolled but 11 patients were excluded because of incomplete investigations. Out of 327 patients 215 (67.7%) were females with the mean age of 57.2 years (SD ±16.7 years). The majority had type 2 DM 72.8%, were urban based 58.7%, aged >60 years 50.5%, and were either overweight 38.5% or obese 32.1% and hypertensive 78.3%. Few patients had shorter duration of DM (36.4%) ranging from 1 to 7 years. A total of 231 (70.6%) cases were taking oral hypoglycemic agents (OHGA) and 96 (29.4%) were on insulin. As regards the laboratory investigations a total of 90.6% had elevated HbAIC of >7%, 74.0% had elevated low-density lipoproteins cholesterol (LDL-c), 45.6% had elevated total cholesterol (TC), and 10% had elevated creatinine. See Tables 1 and 2.

The overall prevalence of DPN was 72.2 % (n=236), males were 67.9%, and females were 74.4%, respectively. A high prevalence was obtained in patients aged above 60 years 84.8% (p= <001), being overweight 77% and obesity 83.8% (p=<0.001), and in those using OHGA 77.5% (p=< 0.004). In addition, a high prevalence of DPN was found in patients with HTN 81.2% (p=<0.001) and with DM duration >7 years >70% (p=<0.011). The majority of type 2 DM patients with DPN 75.3% were using OHGA treatment, in which 135 (78.9%) were on treatment for duration ≥10 years (p =>0.009) with 1.9 OR and 95% CI (1.2 to 3.2). This implies that those type 2 diabetic patients with the DM for more than10 years had 2 times greater risk of being affected with DPN than those with type one. See Table 3.

**Figure 1:**
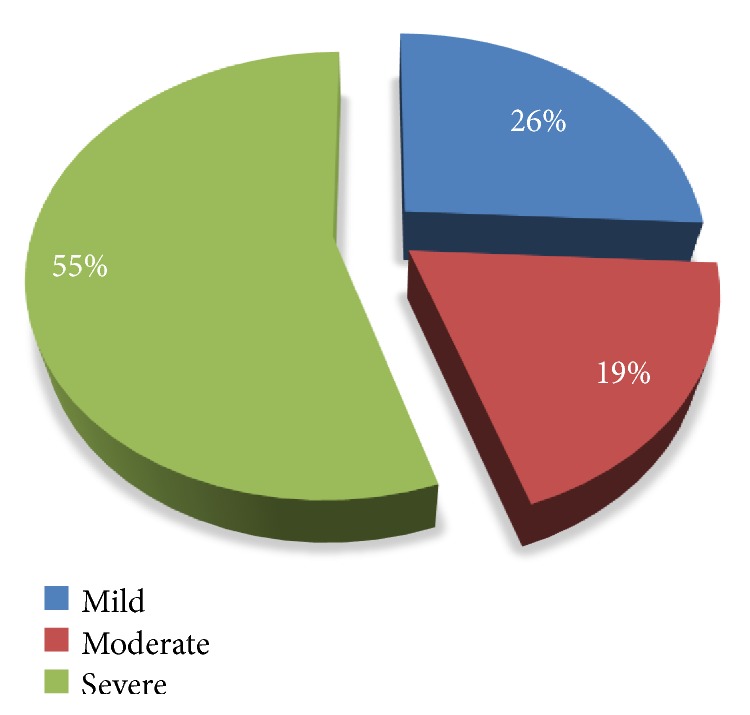
Distribution of severity of neuropathy assessment.

The DPN and its severity were assessed using TCSS, so out of a total of 237 DM patients with DPN had neuropathy, 55% were severe, 19% were moderate, and 26% were mild (Figure 1). The severity was associated with increase in age (p=<0.001), increase in BMI (p= <0.001), and increase in DM duration above 7 years (p= <0.006) but was not associated with a unit increase in HbA1C (p=0.607). The highest risk factors for DPN were age 40-60 years, OR= 6.4 (2.9-14), >60 years 15 (6.8-34.4), BMI 15.1 (3.1–73.6) and 23.3 (4.62–117.5) for overweight and obesity, respectively, OHGA 2.3 (1.4–3.9), and HTN 6.7 (3.8-11.8). Another factor which was associated with DPN is DM duration >7 years 1.4 (0.8-2.4) and 3.5 (1.6-7.8) for categories 8-14 and 15-21 years, respectively, and lastly dyslipidemia (TC) is also associated with DPN 1.2 (1.0-1.4). Surprisingly, HbA1C was not associated with DPN 0.6 (0.2–1.6), and this can be due to effect of categorization. A total of 3% of the study participants had active leg ulcers. See Table 4.

## 4. Discussion

The main outcome of this study was the high overall prevalence of DPN in a population of patients with confirmed diabetes mellitus attending a consultant hospital clinic in Northern Tanzania. The study used a validated bedside clinical tool to document the presence of DPN and measure its severity. The demographic pattern at a referral consultant hospital suggests a cohort of patients with established DM, a possible referral bias with more serious disease, and an overrepresentation of type 2 diabetes. This study has showed a high rate of comorbidities with more patients being overweight/obese, hypertension, and having hyperlipidemia, with 10% evidence of renal impairment and 3% with active diabetic foot ulcers which might be explained by complications of both peripheral vascular disease and peripheral neuropathy. Persistent hyperglycemia as evidenced by elevated HbA1C was almost universal in the study. There are some studies which show nearly similar prevalence as the current study, such as studies from Turkey [12] with DPN of 60%, Yemen [13] with prevalence of 56.2%, and Ethiopia [11]. The current study showed higher degree of DPN when compared to other studies from Uganda [14], South Africa [15], and UK and Germany [16]. The higher difference can be due to different settings, different tool used to determine the DPN, and different population involved, and, lastly, the study was done in a tertiary hospital; hence patients were referred with diverse complications and late diagnosis of diseases [17–21].

The current study showed that half of the patients with DPN had severe form, of which the patterns of severity have a linear correlation with age, BMI, and duration as classified from our findings, but not with their HbA1C. A similar lack of correlation has been reported by Gill HK et al., who proposed that any level of increased glucose beyond the normal level will predispose to neuropathy, and not necessarily a linear correlation [17, 22–24]. Having documented and outlined some of the main problems in persons with DM and DPN in Africa, the study begs the question of the role of prevention for both risk factors and complications. Prevention of microvascular and macrovascular complications involves glucose reduction and long-term glycaemic control and the early initiating of antidiabetic treatment, lowering blood pressure and serum cholesterol, and lifestyle modification [25, 26]. Limitations of the effectiveness of these interventions at the population level in Africa are many because many diabetes cases remain undiagnosed and adherence/compliance to treatment is typically low for practical and financial reasons. Africa simply cannot afford DM or its complications [27–29].

Our study is among the first hospital-based cross-sectional studies to assess the prevalence of diabetic peripheral neuropathy, its severity patterns, and the associated factors in Northern Tanzania. In addition to that, this study is the first to use the simple and quick validated TCSS score to determine the neuropathy and its patterns of severity in Tanzania as a whole.

### 4.1. Study Limitation

This study cannot be generalized to all diabetic patients in Moshi or Tanzania as a whole since it is a referral tertiary hospital where many patients are very sick with multiple comorbidities. Lack of Electro-Diagnostic methods (NCV) which are the definitive and standard techniques for detecting neuropathy can change the trend of these presentations. Lastly, other uncommon causes of neuropathy like vasculitis and vitamin B deficiency were not excluded.

## 5. Conclusion

DPN is widely prevalent in our setting occurring in more than half of the patients attending the diabetes clinic, with more than a half experiencing the severe form. The main associated factors are age, increased BMI, duration of DM, HTN, and OHGA. Having documented these findings, a greater effort should be made to decrease the frequency and severity of PDN in DM patients by education emphasizing daily foot care, tight glycemic control, lowering BP, and lifestyle modification. In addition to this, a simple bedside screening tool can now be used in Africa to diagnose the presence of DPN and assess its severity in patients with DM.

## Figures and Tables

**Table 1:** Demographic characteristics of participants (n=327).

Characteristics	N	%
Age (Year), Mean (SD)	57.2 (16.7)	
*Age, category (Years)*		
<40	41	12.5
40-60	121	37.0
>60	165	50.5
*Sex*		
Male	112	34.3
Female	215	65.7
*Diabetic type*		
Type 1	89	27.2
Type 2	238	72.8
*BMI Category*		
<18.5 kg/m^2^ Underweight	11	3.4
18.5-24.9 kg/m^2^ Normal	85	26.0
25.0–29.9 kg/m^2^ Overweight	126	38.5
≥ 30 Obesity	105	32.1
*Duration of DM (Years)*		
1-7	119	36.4
8-14	114	34.9
15-21	63	19.3
22-28	17	5.2
29-35	10	3.1
36-42	4	1.2
*Residence*		
Rural	135	41.3
Urban	192	58.7

**Table 2:** Medical and physical profiles among diabetic peripheral neuropathy patients.

Characteristics	N	%
*Treatment*		
Oral Hypoglycemic Agent	231	70.6
Insulin	96	29.4
*HbAIC *		
Normal	30	9.2
Abnormal	297	90.8
*LDL-C*		
Normal	83	26.0
Abnormal	236	74.0
*TC (n=320)*		
Normal	174	54.4
Abnormal	146	45.6
*Creatinine*		
Normal	288	90.0
Abnormal	32	10.0
*Hypertension*		
Yes	256	78.3
No	71	21.7
*Diabetic Foot Ulcer*		
Yes	10	3.1
No	317	96.9
*Pattern of DPN*		
None	91	27.8
Mild	61	18.7
Moderate	45	13.8
Severe	130	39.7

**Table 3:** Prevalence of peripheral neuropathy by independent variables (n=327).

Characteristics	Presence of DPN		*P* values
	No, n (%)	Yes, n (%)	
*Age (Years)*			
<40	30 (73.2)	11 (26.8)	
40-60	36 (29.8)	85 (70.2)	.001*∗*
>60	25 (15.2)	140 (84.8)	
*Sex*			
Male	36 (32.1)	76 (67.9)	.2
Female	55 (25.6)	160 (74.4)	
*Residence*			
Rural	38 (28.1)	97 (71.9)	.9
Urban	53 (27.6)	139 (72.4)	
*BMI Category*			
<18.5 kg/m^2^ Underweight	9 (81.8)	2 (18.2)	
18.5-24.9 kg/m^2^ Normal	36 (42.4)	49 (57.6)	.001*∗*
25.0–29.9 kg/m^2^ Overweight	29 (23.0)	97 (77.0)	
≥ 30 Obesity	17 (16.2)	88 (83.8)	
*Treatment*			
Insulin	38 (40.4)	56 (59.6)	
OHGA	52 (22.5)	179 (77.5)	.004*∗*
Both	1 (50.0)	1 (50.0)	
*Diabetic Foot Ulcer*			
Yes	-	10 (100)	
No	91 (28.7)	226 (71.3)	.04*∗*
*Hypertension*			
Yes	48 (18.8)	208 (81.2)	
No	43 (60.6	28 (39.4)	.001*∗*
*HbA1C (%)*			
**<7**	6 (20.0)	24 (80.0)	.315
**≥7**	85 (28.6)	212 (71.4)	
*DM Duration (time) (Range:1,42)*			
1-7	44 (37.0)	75 (63.0)	
8-14	34 (29.8)	80 (70.2)	
15-21	9 (14.3)	54 (85.7)	
22-28	2 (11.8)	15 (88.2)	.011*∗*
29-35	2 (20.0)	8 (80.0)	
36-42	-	4 (100)	

**Table 4:** Logistic regression of potential risk factors associations with DPN (n=327).

Variables	With DPN			
	COR (95% CI)	*P* value	AOR (95% CI)	*P* value
*Age *				
<40	Ref		Ref	
40-60	6.4 (2.9-14)	<0.001	2.7 (1.0-7.4)	0.051
>60	15 (6.8-34.4)	<0.001	5.5 (1.9-15.9)	0.002
*Sex*				
Male	Ref			
Female	1.4 (0.8-2.3)	0.210		
*Residence*				
Rural	Ref			
Urban	1.0 (0.6-1.7)	0.914		
*BMI Category*				
<18.5 kg/m^2^	Ref		Ref	
18.5-24.9 kg/m^2^	6.1 (1.2 - 30.1)	.026	2.3 (0.4-13.8)	0.358
25.0–29.9kg/m^2^	15.1 (3.1 – 73.6)	0.001	3.8 (0.6-23.5)	0.150
≥ 30 Obesity	23.3 (4.62 – 117.5)	<0.001	4.9 (0.8-31.3)	0.089
*Treatment*				
Insulin	Ref		Ref	
OHGA	2.3 (1.4 – 3.9)	0.001	1.1 (0.5-2.3)	0.892
*HbA1C (%)*				
<7 %	Ref			
≥7%	0.6 (0.2 – 1.6)	.319	0.6 (0.2-1.9)	0.385
*HTN*				
No	Ref		Ref	
Yes	6.7 (3.8-11.8)	<0.001	3.8 (2.0-7.2)	<0.001
*DM duration*				
1-7		Ref		
8-14	1.4 (0.8-2.4)	0.248	1.0 (0.5-1.9)	0.953
15-21	3.5 (1.6-7.8)	0.002	2.1 (0.9-5.1)	0.104
22-28	4.4 (1-20.2)	0.056	2.6 (0.5-13.5)	0.264
29-35	2.3 (0.5-11.5)	0.294	1.0 (0.1-5.9)	0.970
36-42	-	0.999	-	0.999
TC	1.2 (1.0-1.4)	0.061	1.1 (0.9-1.3)	0.632

## Data Availability

The data used to support the findings of this study are available from the corresponding author upon request.

## Abbreviations

DPN: Diabetic Peripheral Neuropathy
KCMC: Kilimanjaro Christian Medical Center
DM: Diabetes Mellitus
KCMUCo: Kilimanjaro Christian Medical University College
TCSS: Toronto Clinical Scoring System
JNC: Joint National Committee
BMI: Body Mass Index.
